# Chronic subdural hematoma that may be caused by nephrotic syndrome: a case report and literature review

**DOI:** 10.3389/fneur.2024.1454361

**Published:** 2024-08-22

**Authors:** Hang Xue, Kun Xue, Xiaohui Wang, Weidong Xu, Weitao Zhang, Guangwen Xia

**Affiliations:** ^1^Department of Neurosurgery, Yantaishan Hospital Affiliated to Binzhou Medical University, Yantai, Shandong, China; ^2^Department of Nephrology, Yantaishan Hospital Affiliated to Binzhou Medical University, Yantai, Shandong, China; ^3^Department of the First Neurosurgery, The First Affiliated Hospital of Henan Polytechnic University, Jiaozuo, Henan, China; ^4^Department of Neurotraumatic Surgery, The First Hospital of Jilin University, Changchun, Jilin, China

**Keywords:** nephrotic syndrome, chronic subdural hematoma, mechanisms, surgery, drugs

## Abstract

**Background:**

Chronic subdural hematoma (CSDH) is a common complication of neurosurgery. Craniocerebral trauma is the likely cause. There are no reports relating CSDH with nephrotic syndrome. Its pathogenesis is very rare, and there are no previous reports on treatments for this disease. We report a case of chronic subdural hematoma that may be caused by nephrotic syndrome and review the previous literature on this subject.

**Case summary:**

We report a rare case of chronic subdural hematoma that may be caused by nephrotic syndrome. After the patient was admitted to the hospital, relevant laboratory tests were conducted, and a large amount of protein was detected in the patient’s urine, indicating hypoproteinaemia and hyperlipidemia. The patient was diagnosed with nephrotic syndrome. After the exclusion of related surgical contraindications, the patient underwent trepanation and drainage of the chronic subdural hematoma. Subsequent treatment with oral atorvastatin was provided after surgery. The patient was transferred to the nephrology department for further treatment of nephrotic syndrome if his neurological condition improved. No neurological sequelae were detected at the follow-up visit 3 months after the operation.

**Conclusion:**

Chronic subdural hematomas are rarely caused by nephrotic syndrome. Trepanation and drainage may be considered for patients confirmed to have adequate hematoma liquefaction on imaging and who can tolerate craniotomy. Atorvastatin should be supplemented as prophylactic treatment after the operation. Nephrotic syndrome should be treated as soon as the patient’s neurological condition is stable.

## Introduction

Chronic subdural hematomas rarely develop as a result of nephrotic syndrome, and there have been no reports worldwide. Surgery is the main treatment method. When hematoma liquefaction is sufficient, trepanation and drainage of chronic subdural hematomas should be performed. When hematoma liquefaction is incomplete or trepanation and drainage fail, craniotomy should be performed to clear the hematoma. Postoperative oral atorvastatin is necessary. Nephrotic syndrome should be actively treated after the patient’s neurological condition is stable.

## Case report

**Figure 1:**
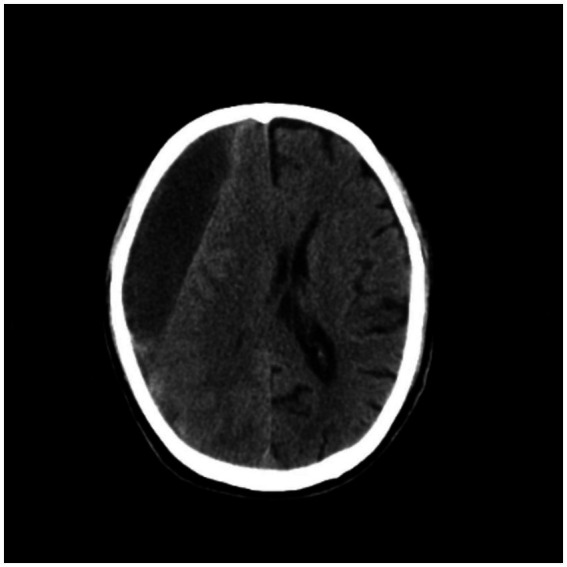
Preoperative brain CT revealed a crescent-shaped low-density shadow in the right frontotemporal parietal area, compression of the lateral ventricle, and a 0.8 cm shift to the left of the midline structure.

**Figure 2:**
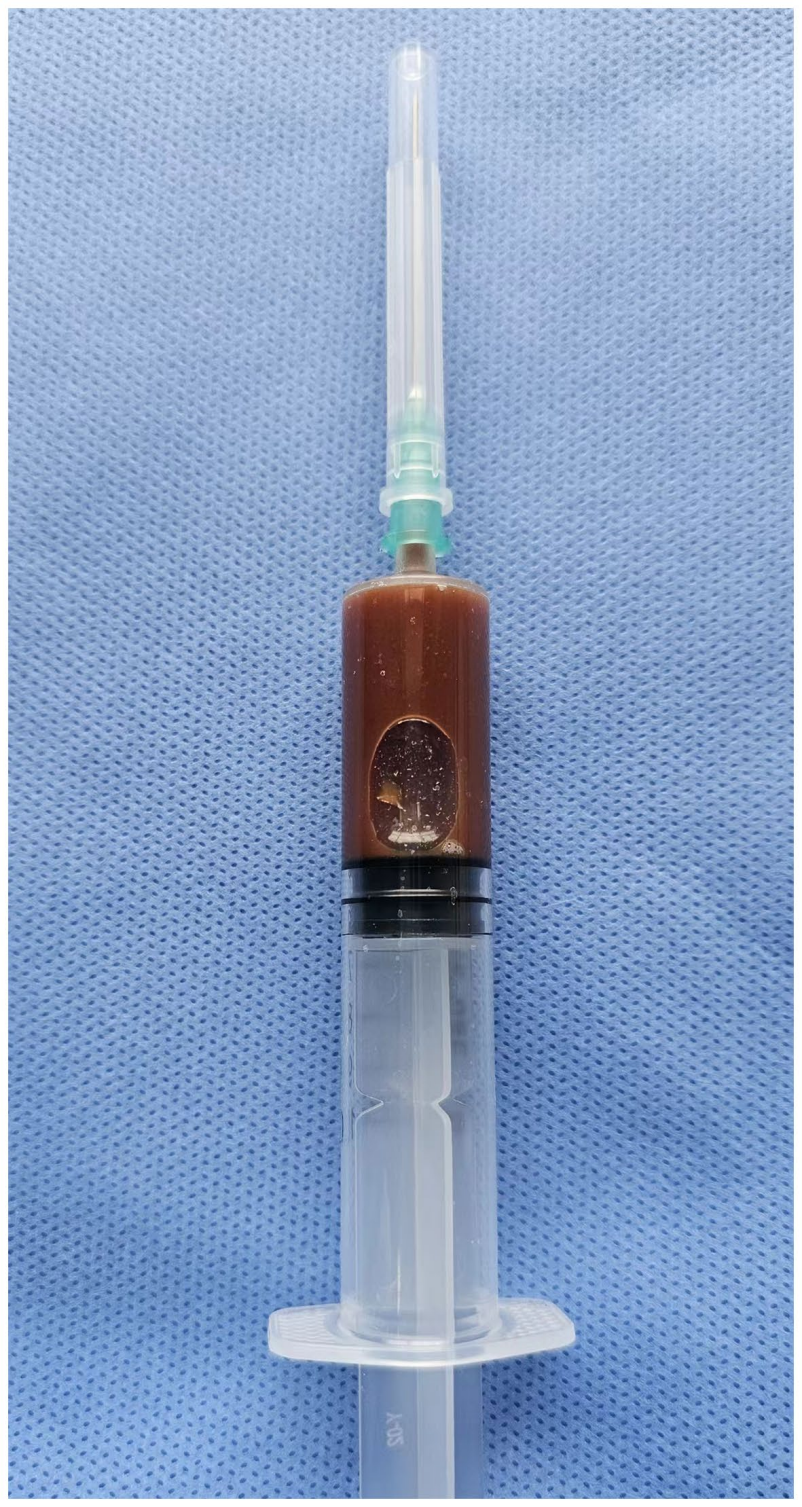
The subdural effusion fluid was thicker, with a large amount of flocculent materials.

**Figure 3:**
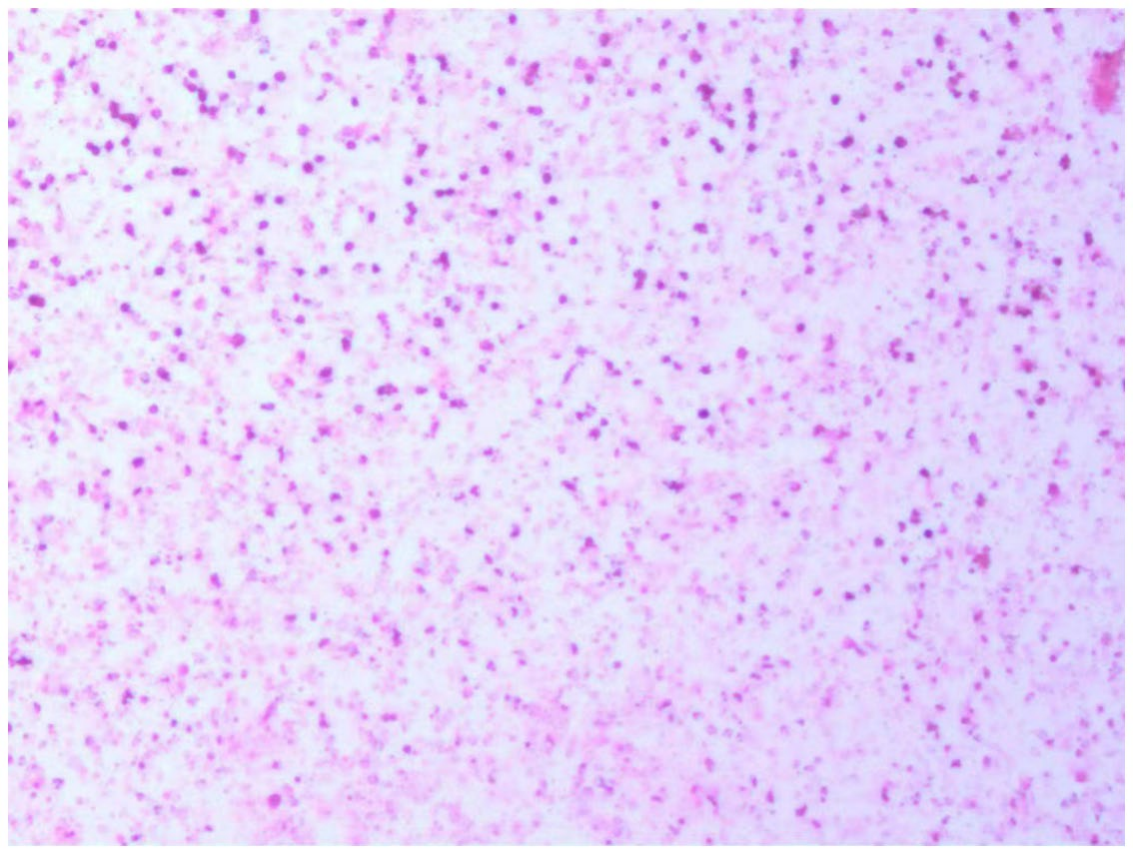
Humoral cytology indicating grade I. A few red blood cells and lymphocytes are visible in the picture, and no dyskaryotic cells are visible.

**Figure 4:**
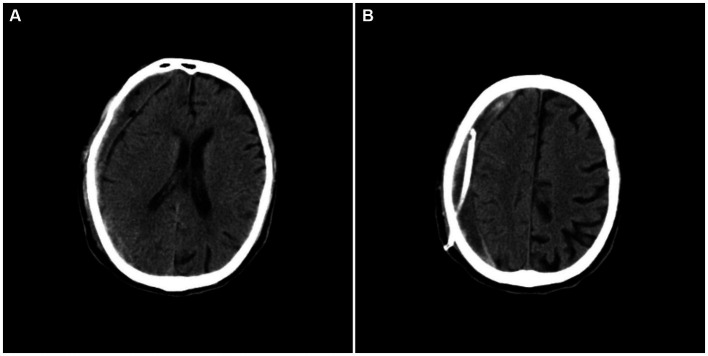
Postoperative craniocerebral CT revealing that the subdural hematoma was smaller in size, the compression of the lateral ventricle was relieved, and the degree of displacement of the midline structure was reduced.

A 77-year-old male patient who experienced exacerbated weakness of the bilateral lower limbs for 1 day after experiencing bilateral lower limb weakness for 3 months was admitted to the Neurosurgery Department of Yantaishan Hospital, Yantai City, on January 14, 2024, for treatment. On admission, physical examination revealed that the patient had clear consciousness and was expressive. The bilateral pupils were 3.0 mm in diameter and sensitive to direct and indirect light. The muscle strength of both upper limbs was grade 5, and that of both lower limbs was grade 4. Muscle tension was normal. The bilateral Babinski sign was negative. The patient reported that he was in good physical health in the past. Preoperative brain CT at admission revealed a crescent-shaped low-density shadow in the right frontotemporal parietal area, compression of the lateral ventricle, and a 0.8 cm shift to the left of the midline structure (Figure 1). Magnetic resonance angiography revealed no significant abnormalities. Magnetic resonance angiography show no spinal dural leakage on thoracic and lumbar MR images. No abnormalities were found on CT scan of the lungs. The patient’s D-dimer level at admission was 14.19 mg/L, and his 24-h urinary protein concentration was 10903.65 mg. The serum albumin concentration was 23.1 g/L. The total cholesterol level was 6.29 mmol/L, and the LDL level was 4.96 g/L. The classification of nephrogenous albuminuria was as follows: urinary immunoglobulin G > 58.6 mg/L, urinary β_2_-microglobulin >4.12 mg/L, urinary microalbumin >348 mg/L, urinary α_1_ microglobulin >110 Mg/L, urinary α_2_ macroglobulin >18.10 mg/L, and urinary transferrin >15.7 mg/L. Antinuclear antibodies, antiextractable nuclear antigen antibodies, antiphospholipase A2 receptor antibodies, and humoral immunity were not abnormal. Subsequently, trepanation and drainage of chronic subdural hematomas were performed. During the operation, the subdural fluid was viscous and contained more floccules (Figure 2). Figure 3 shows that the humoral cytology results were grade I. A few red blood cells and lymphocytes could be seen in the images, and no dyskaryotic cells could be seen (Figure 3). Routine tests of subdural effusion fluid revealed a white blood cell count of 98000×10^6^/L, an 88% composition of multinuclear leukocytes and a positive protein quality (+++). Postoperative CT reexamination revealed that the size of the original subdural hematoma was smaller than that in the preoperative period, the compression of the lateral ventricle was relieved, and the degree of displacement of the midline structure was less than that in the preoperative period (Figure 4). The patient was advised to continue his treatment in the Department of Nephrology after surgery. The patient was followed up by telephone 3 months after surgery, and no residual neurological sequelae were detected.

## Discussion

Chronic subdural hematomas are common complications of neurosurgery and are more common in older men. The overall incidence of CSDH ranges from 1.72 to 20.6 per 100,000 persons per year (1). At present, head trauma is believed to be the factor most likely to contribute to the formation of a chronic subdural hematoma (2), but our patient had no history of craniocerebral trauma. Other rare causes include malignant tumors (3), hematological disorders (4), and spinal dural leakage (5). No related malignancies, hematological abnormalities, or spinal dural leakage on thoracic MRI or lumbar MRI were found in our patient before the operation. After the above etiology was excluded, nephrotic syndrome was considered the likely cause of the disease. There have been no reports worldwide thus far, and there are only a few reports of cerebral hemorrhage caused by nephrotic syndrome. The likely cause of this disease has yet to be determined.

Hu P noted that hypoalbuminemia directly affects red blood cell deformability and possibly affects endothelial function (6). According to the Cox proportional hazards model conducted by Seliger and colleagues, a 1-g/dL decrease in the serum albumin concentration was associated with a 43% increased risk of stroke among patients with end-stage renal disease (7). Our patient’s serum albumin concentration was 21.3 g/L, which indicates hypoproteinaemia. This factor may be involved in the occurrence and development of the disease.

In 2010, Solak et al. suggested that nephrotic syndrome may be secondary to amyloidosis (8), which is a common cause of cerebral hemorrhage. This finding is consistent with the conclusion of the study performed by Niculae (9) in 2017. These authors suggested that small blood vessel damage caused by amyloidosis immune deposition is a common cause of cerebral hemorrhage. Notably, amyloidosis often leads to lobar hemorrhage; however, there are no reports related to chronic subdural hematoma. However, in 2013, Kitamura et al. reported that an acquired factor V inhibitor is a rare bleeding disorder that is known to be difficult for physicians to treat because of their limited knowledge and uncertain relationship with autoimmune disease (10). They suggested that acquired factor V inhibitors can cause both nephrotic syndrome and intracranial hemorrhage (10). However, owing to limited knowledge and its uncertain relationship with autoimmune diseases, additional studies are needed.

Considering the pathogenesis of our case, the possible mechanism is as follows: (1) Amyloidosis occurs in both the kidney and brain. The medial and outer membranes of intracranial blood vessels are replaced by amyloid protein. Abnormal vascular endothelium increases the permeability of blood vessels; thus, various plasma components, including proteases, can invade the blood vessel wall, resulting in intimal hyalinoid thickening. The blood vessel wall contains fibrinoid necrotic components, which increase the fragility of blood vessels and are prone to rupture and bleeding. (2) Some specific types of nephrotic syndrome are associated with acquired FV inhibition, and these coagulation disorders may be the cause of intracranial hemorrhage. Damage to small blood vessels is aggravated by hypoproteinaemia-induced red blood cell deformability and possible endothelial dysfunction. Notably, the continuous accumulation of blood in the subdural space induces chronic inflammation and the formation of highly permeable new capillaries (11), which may also be involved in the further development of this disease.

There are two main surgical treatment methods for chronic subdural hematoma: trepanation and drainage, as well as craniotomy for hematoma evacuation. Owing to their long-term clinical applications, the safety and effectiveness of trepanation and drainage for chronic subdural hematoma have been widely recognized. These are currently the most widely used methods (12, 13). Craniotomy for hematoma evacuation followed by the placement of a large bone flap is rare because of the high risks of trauma and postoperative complications (14). However, nephrotic syndrome puts the human body in a state of hypercoagulation, so the hematoma is more likely to coagulate and form massive floccules that block the drainage tube, which may lead to poor results or even failure of trepanation and drainage. Therefore, the authors suggest that patients who have adequate liquefaction of chronic subdural hematomas and can tolerate craniotomy should undergo trepanation and drainage. If the drainage effect is poor and liquefaction of the hematoma is not obvious, craniotomy is recommended to clear the hematoma if the patient can tolerate it.

In a preliminary study, an inhibitor of 3-hydroxy-3-methylglutaryl-coenzyme A reductase was first reported to reduce the hematoma volume in CSDH patients (15). Atorvastatin inhibits inflammatory angiogenesis and promotes vascular maturation by reducing macrophage infiltration and downregulating vascular endothelial growth factor, monocyte chemotactic-1, tumor necrosis factor-α and transforming growth factor-β_1_ (16). And previous research has shown that atorvastatin is an effective conservative therapy of chronic subdural hematoma (17). After surgery, our patient was treated with oral atorvastatin.

Nephrotic syndrome is likely to recur if it is ineffectively treated with surgery. Recurrence is considered related to excessive fluid leakage due to increased capillary permeability caused by hypoproteinaemia. Therefore, the treatment of nephrotic syndrome is crucial.

In summary, chronic subdural hematomas caused by nephrotic syndrome are extremely rare and have no typical clinical manifestations. The following characteristics often indicate the possibility of chronic subdural hematoma caused by nephrotic syndrome: (1) Severe proteinuria and decreased serum albumin concentrations are present. (2) In addition to neurological symptoms, there are clinical manifestations such as edema, susceptibility to infection, oliguria, and anuria. (3) Chronic subdural hematoma without a history of trauma. When the above characteristics appear, the possibility of chronic subdural hematoma caused by nephrotic syndrome should be considered.

## Data availability statement

The raw data supporting the conclusions of this article will be made available by the authors, without undue reservation.

## Ethics statement

The studies involving humans were approved by the Yantaishan Hospital Affiliated to Binzhou Medical University. The studies were conducted in accordance with the local legislation and institutional requirements. Written informed consent for participation in this study was provided by the participants’ legal guardians/next of kin. Written informed consent was obtained from the individual(s) for the publication of any potentially identifiable images or data included in this article.

## Author contributions

HX: Writing – original draft, Writing – review & editing. KX: Writing – review & editing. XW: Writing – review & editing. WX: Writing – review & editing. WZ: Writing – review & editing. GX: Funding acquisition, Resources, Writing – original draft, Writing – review & editing.
